# Clarifying the role of clinical supervisors according to physiotherapists at a higher education institution

**DOI:** 10.4102/sajp.v75i1.523

**Published:** 2019-06-18

**Authors:** Taryn-Lee Voges, Josè M. Frantz

**Affiliations:** 1 Department of Physiotherapy, University of the Western Cape, Cape Town, South Africa; 2 Faculty of Community and Health Sciences, University of the Western Cape, Cape Town, South Africa

**Keywords:** clinicians, clinical education, clinical supervisors, higher education, roles

## Abstract

**Background:**

The roles of doctors and nurses in clinical supervision and clinical education are well defined. The role of the physiotherapist in clinical education has not been clearly defined.

**Objectives:**

The aim of this study was to define and clarify the views and experiences of physiotherapy clinical supervisors regarding clinical education and their role in contributing to student learning.

**Methods:**

This qualitative exploratory study targeted 17 physiotherapy clinical supervisors, employed in a physiotherapy department, at a local university in the Western Cape. Twelve of the 17 participants agreed to participate in the study. Data were collected by means of in-depth audio-taped interviews, at a time convenient for the participants. Each transcript was read individually by the first author and notes made in the margins to highlight interesting concepts that emerged. The different types of concepts were listed and categorised, while common categories were grouped into themes.

**Results:**

Based on the results, the clinicians’ role is viewed as a valuable asset in clinical education, embodying the role of an educator, mentor, role model and communicator. Clinical supervisors discussed their roles in terms of understanding the importance of clinical supervision, providing guidance within a clinical setting, role modelling and professionalism. Although clinical supervisors play a significant role, they experienced a few challenges including role clarification and students’ lack of knowledge.

**Conclusion:**

This study highlights that clinical supervisors and clinicians fulfil significant roles in assisting students to integrate theoretical and clinical knowledge.

**Clinical implications:**

Understanding the expectations of clinical supervisors in supporting clinical education is important for higher education institutions and the clinical sector.

## Introduction

In our current health care environment, the role of the health professional does not necessarily only focus on the care of patients, but includes leadership, research, administration and clinical supervision. Although the concept of clinical supervision is a term that is commonly used in health and social care professions, its definition may vary. Therefore, the understanding of clinical supervision internationally, and in the physiotherapy profession, may be different from the local context. The Chartered Society of Physiotherapy (2000) perceives clinical supervision as a process that will play a key role in the future development of local, robust systems of continuing professional development (CPD). Sellars (2004:74) concurs that ‘clinical supervision provides the opportunity for a formal system of professional support and development, which is valued by individuals engaged in the process’. According to the Australian Department of Health (2005), clinical supervision is defined as ‘the process of two or more professionals formally meeting to reflect and review clinical situations, with the aim of supporting the clinician in their professional environment’.

However, in a study conducted in the United Kingdom, by Hall and Cox (2009), the authors highlight that clinical supervision is understood as one aspect of CPD. In this study, the authors explained that clinical supervision is a familiar term for physiotherapists, in the context of workplace learning for undergraduate students, as well as an activity that occurs with senior colleagues, to develop clinical reasoning and support. Therefore, this definition closely defines the way it is understood in the South African context. In the South African context, the role of the health professional in the clinical setting is linked to the provision of quality undergraduate education to health professional students, through clinical supervision.

Among physiotherapists in South Africa, the term ‘clinical supervision’ refers to the clinical education of students. According to Schoen et al. (2008:263), ‘Clinical education enables physiotherapy students to acquire information, skills as well as competencies necessary for clinical practice, to gain confidence in their clinical reasoning and application of theoretical knowledge’. Therefore, the belief underpinning clinical supervision is learning from practice. In the South African context, clinical supervisors are employed by the university to facilitate this learning and critical thinking, as well as to teach and evaluate the clinical performance of students. Keiller and Hanekom (2013) explain that critical thinking is:

the ability of a clinical supervisor to analyse complex data and situations in order to implement appropriate strategies or actions that are necessary for effective problem-solving and decision-making in the clinical arena. (p. 11)

The role of clinicians, as educators and supervisors, has been previously explored by Manninen et al. (2015). The authors highlight that the supervisors’ pedagogical role is to facilitate student learning, and therefore they should assist in seeking learning opportunities for students to practise their clinical skills. Consequently, it is understood that supervisors should aim to facilitate experiential learning (learning by doing) among students. In a study conducted by Jokelainen et al. (2011), the results revealed that the mentoring of students integrates individual, organisational aspects with environmental, collegial, pedagogical, as well as clinical attributes in placements. According to the literature, significant progress is evident among doctors (Razmjou et al. 2015) and nurses (Bos, Silèn & Kaila 2015) in this area.

Archer (2011) highlights that while health professionals, acting as supervisors, may be the experts in their fields, they do not always have the necessary teaching skills. Undergraduate programmes for several disciplines require clinical supervisors to teach students in clinical settings. Physiotherapy undergraduate programmes, offered across all the universities in South Africa, employ clinical supervisors, who are required to educate students in clinical settings. Even though clinical supervisors are employed by universities, it is the universities’ responsibility to ensure that all clinical supervisors receive adequate support and opportunities to improve their skills in supervising. The findings of a study, conducted by Archer (2011), revealed that:

although most clinical teachers are enthusiastic and take their role as teachers of future generations of healthcare professionals seriously, they often lack knowledge of educational principles as well as teaching strategies, and thus may be inadequately prepared for this additional professional role. (p. 6)

Therefore, building the capacity of all health professionals, to empower them for their various roles, is essential.

In South Africa, the relationship between health institutions and universities is essential for the training of students. However, the relationship needs to be beneficial to both parties, hence the importance of capacity building for clinicians, who also serve as clinical supervisors. In developing countries, capacity building has become a popular strategy to develop sustainable human resources for health (Kumar, Adhish & Deoki 2014).

However, before intervention strategies can be designed, it is important to understand the views of the key stakeholders, and in this case the views of the physiotherapy clinical supervisors employed by the higher education institutions. A systematic review, conducted by Farnan et al. (2012), explored the effects of clinical supervision on patient and residency education outcomes. Its findings highlighted that when clinical supervision is improved, both patient- and education-related outcomes are improved. Consequently, if both patient and education outcomes are to be achieved, a more systematic collaboration between the clinical settings and the university is necessary (Bos et al. 2015).

McKimm and Swanwick (2011) highlight that if the importance of the role of clinical supervision in clinical education is to be acknowledged, there needs to be a change in culture. According to physiotherapists, a culture needs to be fostered, which aims to encourage all physiotherapists to contribute to this vital process of clinically educating undergraduate students, to develop competent entry-level physiotherapists. According to Schoen et al. (2008), clinical education enables students to integrate the required knowledge, skills and behaviour, which leads to the development of entry-level physiotherapists who act with professionalism, competence and leadership. In addition, if clinical supervisors value the contribution they make in the process of clinical supervision, the impact will be far reaching (Schoen et al. 2008).

The concept of clinical supervision in clinical education may vary among health professionals; therefore, it is important to have a better understanding in the physiotherapy arena. If clinicians and clinical supervisors are unsure of their role in clinical settings, how do they expect students to develop into qualified professionals? Therefore, the first author explores the clinical supervisors’ views on how they perceive their contribution to student learning, to obtain a clearer understanding. The aim of this study was to define and clarify the views and experiences of physiotherapy clinical supervisors regarding clinical education and their role in contributing to student learning.

## Methods

The first author used a qualitative exploratory research design for this study. Purposive sampling was used for the selection of participants based on their suitability for the study and their knowledge of the specific phenomenon (Cozby 2012). The population consisted of 17 physiotherapy clinical supervisors, employed in a local university’s physiotherapy department, for the 2014–2015 academic period. Twelve of the 17 participants agreed to participate in the study.

Data were collected by means of face-to-face, in-depth, semi-structured interviews, which were audio-recorded by the first author, at a time convenient for the participants. The interviews allowed for in-depth exploration of clinical supervision exposure, views about the process, how equipped the participants were to be clinical supervisors, as well as encouragement of student learning. Prior to starting the interview, informed written consent was obtained from the participants. The first author started the interviews with a pre-interview discussion and introductory comments, before introducing the first question, to encourage the participants to share their perspectives and experiences (Zikmund et al. 2009). The interview was initiated with a broad question: ‘What do you think your role is, as a clinical supervisor in clinical education?’ Subsequently, the first author used probes to facilitate deeper discussion.

Tesch’s eight steps of qualitative data analysis are outlined by Creswell (2009), namely (1) transcription and checking of data, (2) immersion in transcribed data, (3) creating codes, (4) identify themes, (5) creating sub-themes and categories, (6) finalisation of themes and sub-themes, (7) verified process and consensus and (8) recode existing data. The analysis was conducted manually by the first author. Data were transcribed by an individual fully trained and qualified in transcribing qualitative data. Each transcript was read individually by the first author, and notes were made in the margins to highlight interesting concepts that emerged. The different types of concepts were listed and categorised, while common categories were grouped into themes. To ensure that the information gained was accurate during the interview process, the first author concluded and summarised the findings after each question.

Credibility was ensured through the audio-recording and notes taken throughout the interview process. The direct quotations extracted from transcripts indicate confirmability, as the findings did not emerge from the predispositions of the first author but rather from the data provided by participants. The study explicitly reported on the research methods, allowing for transferability. Lastly, accurate and detailed records of research methods and strategies for data collection and analysis were recorded, indicating dependability.

### Ethical considerations

This study was ethically approved in 2014 by the Senate Research Committee at the University of the Western Cape under project number 14/8/10.

## Results and discussion

### Demographic data of participants

In this study, the views and experiences of 12 physiotherapy clinical supervisors were explored. The clinical supervisors were employed by the university to clinically guide and educate students in the clinical settings, at both third- and fourth-year levels. Experience among the participants varied, as both practising physiotherapists and clinical supervisors. Their experience ranged from 1.5 years to 27 years as practising physiotherapists, and from 6 months to 22 years as clinical supervisors. Most of the participants had experience of working at clinical placement as clinicians, prior to being clinical supervisors.

### Themes and sub-themes

Three key themes emerged from the data analysis, namely the role of clinical supervisors in clinical supervision, challenges experienced as a clinical supervisor and the clinician’s role according to clinical supervisors, as shown in Table 1.

**TABLE 1:** Emerging themes and sub-themes.

Themes	Sub-themes
Theme 1: The role of the clinical supervisors in clinical supervision	Educator role Mentor role Clinical supervisors as role models Communicator
Theme 2: Challenges experienced as a clinical supervisor	Role clarification and identification Students’ lack of theoretical knowledge Are clinical supervisors equipped to teach?
Theme 3: The clinician’s role according to clinical supervisors	Understanding the importance of the clinician’s role Clinicians providing clinical guidance in the clinical setting Role modelling and professionalism

### The role of the clinical supervisor

The roles that emerged from this first theme included four sub-themes namely, educator role, mentor role, clinical supervisors as role models and communicator. The participants were of the opinion that, in their specific role of clinical educator, they facilitate learning, which is a form of clinical education.

‘An educating role is fulfilled…I do play a role in educating them because sometimes in the facilitation process you will find they got gaps, so gaps in their knowledge perhaps things that they have been taught… I cannot necessarily re-teach them those things but remind them about things that they were taught.’ (P3, female)

A few participants expressed that too often the clinical supervisors and clinicians refer students back to the literature, ‘to read up’, without an effort to stimulate the thinking processes of the student, to reach the answer. According to Bos et al. (2015:39), the current educational goals of students are different to those of previous years, as students often require qualified support to integrate theory into practice.

‘…students often get this: “go read up”. I will often take the time and say: “okay so this is the patient’s situation, this is the diagnoses, this is how it affects the lungs”…so I do take that time to explain if I see that they just not get it…I will give information if I feel that this was something that was overlooked, I would say go and read up if it was something basic.’ (P4, female)

Evidently, clinical supervision in physiotherapy is a practice that requires the clinical supervisor to supervise and facilitate student learning, through guidance, as well as support in the clinical arena, providing links between theory and practice. These ideas are represented in the excerpt provided below:

‘My role as a supervisor is to guide students and facilitate their learning, and to help them with whatever they learn in class to apply that in a clinical setting. They must bring their theory and bring it in practical use. For some students it is difficult, because they have this theory in their minds so they cannot really select…It’s not like “I’m telling you”… but “let me facilitate your clinical reasoning”. Clinical supervision for me is about teaching them attentiveness and it’s not about them.’ (P7, female)

Likewise, Lekkas et al. (2007) studied graduates’ prize learning from participation in clinical contexts and explained that there is wide acknowledgement that professional skills are crystallised through the integration of theory and practice in the workplace. The role of the educator is revealed as one that aids the students to integrate theory into practice, as well as stimulate the clinical reasoning of the student and to use appropriate teaching strategies. Clinical reasoning is alleged to be a process that needs to be facilitated through linking the boxes, thereby integrating theory into practice. According to Higgs, Jones and Loftus (2008), clinical reasoning can best be developed when students work with clinical educators in the clinical setting. These clinical educators, therefore, act as mentors in the clinical placements.

There is a need for both clinical supervisors and clinicians to realise that students revere their educators, observing their behaviour and attitudes. According to Kilminster and Jolly (2000:834), ‘A teacher’s interpersonal behaviours, planning preparation and the ability to run a session are key factors in good teaching’. Educators should be keen to be good role models and accept the importance thereof. All clinical supervisors and clinicians model behaviour, either good or bad. Ultimately, the student will choose to either mimic the behaviour of the educator or refuse it. Therefore, educators have to be role models that students would be proud to imitate. The desire of clinical supervisors to be good role models is expressed by one participant as presented below:

‘I also want to be a role model for them like when they are watching. They must see if you treat the people with respect it does not matter whether it’s a gangster or a beggar.’ (P8, female)

### Challenges experienced as a clinical supervisor

Although clinical supervisors were able to identify the roles that they fulfilled, they might not have been comfortable with these identified roles. The main challenge identified by clinical supervisors was their role as teachers. The participants questioned their skills as educators, in terms of knowledge and passion, as well as whether it was part of their role. Although being an educator was identified as being part of their role, a few of the participants reported they did not consider teaching as part of their duty. They were of the opinion that students should come prepared with the baseline theory. These opinions are present in the excerpts below:

‘I think I have a limited role in educating them (the students) on theory on anything that we do. I feel like that’s the role of the lecturers at varsity, so my role in teaching is limited to practical…’ (P12, female)

‘I don’t know whether it is my responsibility or not… and yes there is a debate about it, but I would be happy to rather tell the student because then I feel like this is my duty…even if you say to me the university didn’t teach it to me.’ (P4, female)

Razmjou et al. (2015:248) asserted that ‘Medical residents found poor supervisory roles in clarification of their responsibilities and learning goals’. Role clarification is important to progress in physiotherapy clinical education. Clinical supervisors need to ensure that they are like-minded regarding this matter. One party cannot believe that his/her role is not to teach, while the other believes that it is. There should be consistency in placements. Ultimately, the students do not receive the necessary clinical education to become competent, independent practitioners. According to Cross (1995:503), conflicting aims and viewpoints of clinical supervisors may contribute to difficulties during the implementation and quality assessment of effective clinical education programmes.

Another challenge highlighted by the participants was the lack of knowledge of the students. A few participants reported that it was a challenge, when the students lack knowledge of theory. Therefore, time allocated to integrate theory into practice is instead spent on teaching theory. Consequently, the students’ practical skills and clinical reasoning receive minimal attention. The participant explained that to concentrate on the one aspect, without the other, was unacceptable; the foundation theory has to be embedded.

‘It is very difficult because I only see the student one hour per week and it is very difficult because if they don’t have backup (theory) I cannot effectively use that hour. Then you really have to spend that hour on theory, then it is not good…There is really no time to spend on basic theory to teach a student, how to apply and to reason clinical if there is no theoretical back ground…I would refer them back to the faculty…because the university has classes that they can attend to make sure they are in place and work on their basic skills.’ (P8, female)

Similarly, Chipchase et al. (2012) suggest that students are to fully capture clinical opportunities by making sense of their experiences. For this to be achieved health professional curricula must prepare students for clinical learning before they encounter the clinical environment and experiences. Therefore, theory influences clinical learning and experiences, largely benefiting practical learning of clinical students.

### The clinician’s role according to clinical supervisors

The role of the clinician in clinical education was discussed by clinical supervisors, resulting in four sub-themes, namely understanding the importance of the role of the clinician, providing guidance in the clinical setting, role modelling and professionalism.

In identifying the role of the clinician, the supervisors noted that there were overlaps in the roles of supervisors and clinicians. Many participants agreed that the clinician should play a supportive role regarding students. In a study conducted by Edgar and Connaughton (2014), it was established that clinical educators may be supervisors employed by the educational institutions, or clinicians employed in the sector.

However, despite the varied role of the clinical educator, the authors explained that it was not appropriate to add the role of educator to that of clinician. The participants strongly emphasised that the role of the clinician is important in the clinical exposure of the student; however, do they have the time? The clinician has more access to the student; however, do they have the resources to educate the student clinically? Manninen et al. (2015:55) assert that it is common for the supervision of students to be prioritised after patient care, resulting in the lack of allocated resources to clinical supervision of clinical students. These are only a few of the barriers that exist in clinical education.

The participants expressed that clinicians have the ability to provide the student with learning opportunities, as they are in the same environment as the student as presented in the excerpt below. According to Cross (1995), clinicians possess the necessary clinical skills and familiarity of modern equipment necessary for optimal patient care. Cross (1995) continues to argue that clinicians have much to offer clinical students and should be at the forefront and drivers of the clinical education process.

‘I think clinicians has a big role, okay the university itself is obviously the biggest in terms of theory and getting the foundation right, but clinicians definitely play an important role because what happens in the hospital is between the clinician and the student. The decisions that the clinician makes actually impacts on the student whether the students going to like neuro or paeds etcetera.’ (P6, female)

A few participants considered that educating students was not only the role of the supervisor, but the clinician’s role as well. The participants maintained that if the hospitals allow students into the environment, the clinical settings should take some responsibility for educating the students. Manninen et al. (2015:55) assert that while it remains challenging there is a need for clinical practice to create learning environments in which the supervision of students is regarded as equally important. According to the participants, the same applies to clinicians; if their patients are being treated by students, it is their responsibility to supervise the student, to ensure that no harm is done, and the patient is receiving adequate treatment.

‘Clinicians often have a big role in facilitation of learning and I think it’s missed because they more concerned about whether their workload is getting done because they don’t spend as much time with the student; I don’t know what amount of time they should be spending with the student.’ (P2, female)

According to Edgar and Connaughton (2014), clinical educators, namely clinicians and clinical supervisors, typically have the responsibility for graded, or competency-based, assessments of students’ knowledge and skills, as well as the development of professional workplace skills. This idea is expressed by a participant in the excerpt provided below.

‘In terms of assisting the students they should also play a role in checking up and assisting because clinicians give their patients to the student, so it’s also the clinician’s responsibility to make sure that the patient is suitably treated or appropriately treated, so they should follow up on these patients whether the student is doing the right thing and therefore kind of like a safety net for a student.’ (P6, female)

In a study conducted by Kilminister et al. (2007), the findings suggest that helpful supervisory behaviours include giving direct guidance on clinical work, linking theory and practice, engaging in joint problem-solving, offering feedback, as well as reassurance and providing role models. The participants highlighted that clinicians could provide guidance in the clinical setting, in terms of administration, time management and patient management, including discharge planning.

## Conclusion

The aim of this study was to explore the role of the clinical supervisor (employed by the university), as well as the clinical supervisors’ views on the role of clinicians (employed by the medical institution) in clinical education. The authors highlighted that clinical supervisors as well as clinicians fulfil significant roles in integrating theoretical and clinical knowledge. In clinical supervisors’ observations, clinicians contribute in different ways to clinical education than clinical supervisors. These different ways include teaching students how to be professional by role modelling professionalism; teaching them how to complete administration tasks; teaching by engaging them in tutorials, assessments and treatments in authentic learning situations; and providing opportunities of learning to students by attending to their needs. It is highlighted that clinical supervisors would be keen on clinicians arranging more tutorials and treatment sessions with the students to further improve their critical thinking skills, as well as practical techniques and the applications thereof.

Most participants agreed that, collectively, there are overlapping roles to support the students, as both parties could teach and guide, facilitate and demonstrate. The participants considered the clinicians to be clinical supervisors as well; however, with a different influence on student learning. The aim of clinical education is to assist the student to bridge the gap between theory and practice, and between the classroom and the clinical setting. When focusing on integrating theory into practice, students should be guided to understand what knowledge and skills are required during patient management, which could be facilitated by both the clinician and the supervisor.

Although the study highlights the views and experiences of clinical supervisors about clinical education and their contribution towards students, learning, the study is limited by including one institution only. Therefore, the findings are not generalisable. Further exploration including multiple higher education institutions and clinical placements is required. Future research including the views and experiences of clinical supervisors and students is required to obtain a more holistic view. In addition, opportunities need to be created for clinicians to be role models and interact with students, within clinical placements and possibly as guest lecturers at universities. Clinical supervisors need to demonstrate and guide students through appropriate role modelling. Lastly, a partnership needs to exist between the university and clinical placements. The alignment of these institutions, working as ‘one’, creates a positive image and impact on the perceptions and learning of students.
